# Economic evaluation of vaccination against COVID‐19: A systematic review

**DOI:** 10.1002/hsr2.1871

**Published:** 2024-02-07

**Authors:** Dolatshahi Zeinab, Nargesi Shahin, Mezginejad Fateme, Bagheri Faradonbeh Saeed

**Affiliations:** ^1^ Department of Health Policy, School of Health Management and Information Sciences Iran University of Medical Sciences Tehran Iran; ^2^ Department of Health Management and Economics, Faculty of Health Ilam University of Medical Sciences Ilam Iran; ^3^ Department of Hematology, School of Allied Medicine, Cellular and Molecular Research Center Birjand University of Medical Sciences Birjan Iran; ^4^ Department of Health Services Management, School of Health Ahvaz Jundishapur University of Medical Sciences Ahvaz Iran

**Keywords:** cost‐benefit analysis, cost‐effectiveness analysis, cost‐utility analysis, COVID‐19, vaccine

## Abstract

**Background and Aims:**

Coronavirus has burdened considerable expenditures on the different health systems. Vaccination programs, the critical solution against pandemic diseases, are known as safe and effective interventions to prevent and control epidemics. We aimed to perform a systematic review to provide economic evidence of the value of different types of vaccines available to combat the Covid‐19 to all health policymakers worldwide.

**Methods:**

Electronic searches conducted on Medline/PubMed, Cochrane Library, Web of Science, Scopus, Embase, and other economic evaluation databases. Related and published articles searched up to March 2022 by using keywords such as “Vaccination,” “Covid‐19,” “Cost‐benefit,” “Cost‐utility,” “Cost‐effectiveness,” “Economic Assessment,” and “Economic evaluation.” Followed by choosing the most suitable articles according to inclusion and exclusion criteria, data captured and the results extracted. The quality assessment of the articles performed by the checklist of CHEERS 2022. Finally, 13 articles included in the review.

**Results:**

All messenger RNA vaccines were dominant with approximately 70% coverage against no vaccination in the primary vaccination program except in one study that looked at booster effects. From a payer's perspective, a dollar invested in a vaccine would be less profitable than from a societal perspective. Therefore, primary mass vaccination can be considered a cost‐effective intervention in primary vaccination to save more lives and produce more positive externalities. However, the cost‐benefit ratio for all vaccines increases when statistical lifetime value and global economic and educational disadvantages are considered.

**Conclusion:**

The COVID‐19 primary vaccination programs in regional outbreaks, from a long‐term perspective, will demonstrate substantial cost‐effectiveness. It is suggested that due to the positive externalities of vaccination, primary mass vaccination, with the help of COVAX‐19TM, could be considered a reliable way to combat viral epidemics compared to the loss of individual lives and economic and educational disturbances around the world.

## 1 INTRODUCTION

The high transmissible Coronavirus Disease (COVID‐19) as a novel catastrophic pandemic in this century started and became widespread at the end of 2019, made a massive rate of morbidity and mortality in a short period and led to a global public health crisis.^1^, ^2^, ^3^

Subsequently, this situation has made global economic contractions, difficulties and recessions, economic downturns, and crises mainly due to medical expenses and productivity loss worldwide.^3^, ^4^ New research on the Covid‐19 vaccine showed that people attitudes towards the Covid‐19 vaccination, specially the role of potential policy options, characteristics of the vaccine, and disinformation plus misinformation played an important role in people's acceptance of vaccination.^5^ In two recent studies the researcher noted that vaccines with higher efficacy were associated with a greater chance of vaccination acceptance, while emergency use authorization by the Food and Drug Administration^6^, ^7^ or vaccines that required more time for political approval were associated with greater uncertainty.^8^ In another very recent study, the researchers are going to show that risk perception and trust in health institutions are the most relevant predictors of intention to be vaccinated.^9^ Moreover, the COVID‐19 pandemic can lead to ignorance of other life‐threatening diseases and mental disorders caused by people's isolation, as well as social damages from unemployment rate elevation, etc….^10^, ^11^, ^12^ So not only does this global concern threaten the health of communities, but it also jeopardizes the capacity of health systems and the world economy which results in the loss of millions of dollars from governments resources.^10^, ^11^, ^12^, ^13^

Despite severe public health policies that have been implemented in most of the involved countries to prevent COVID‐19 transmission, the pandemic cessation has not entirely succeeded. Therefore, to deal with this problem, it was necessary to identify effective pharmacological interventions and found a more reliable strategy for prevention such as vaccination.^14^ Despite the goals of the Immunization Agenda 2030 (IA2030) campaign to further strengthen global immunization and reduce global disparities, the global COVID‐19 pandemic has led to further disruption of routine immunization and campaign activities. It has been estimated that even if the IA2030 targets were met, the epidemic would have resulted in at least 50% fewer fully vaccinated persons and 5.22% more deaths worldwide.^15^

In this regard, scientists and researchers in different countries attempted to discover an effective vaccine to reduce viral transmissibility and finally control disease consequences.^16^, ^17^ Considering the insufficiency of the available preventive measures, it seems prophylactic vaccination is a cost‐effective and promising remedy provoking immunity by stimulating neutralizing antibody production and memory T lymphocytes to fight the pathogen and diminish disease transmission.^18^

On the other hand, although vaccination may be a critical achievement in this challenging situation to address this health problem, there are concerns regarding the costs of preparation. When dealing with the costs of public vaccination, which is different according to the type of vaccine and estimated to be approximately $50 billion to save the world, usually there is no limitation for high‐income countries, whereas the majority of countries are not able to access sufficient amount especially low and middle‐income countries.^11^, ^19^, ^20^ So, a big dilemma is providing sufficient financial sources to implement vaccination programs for a large number of populations in a short time. Nevertheless, it is supposed that this huge cost prioritizes and prevents more devastating economic impacts of COVID‐19.^21^, ^22^

According to the World Health Organization (WHO) COVID‐19 vaccine tracker and landscape, by the start of April 2022, 153 vaccines are in clinical development and 196 in preclinical development.^23^ Moreover, as of April 4, 2022, more than 11 billion vaccine doses have been administered worldwide to encounter COVID‐19.^24^ So, the availability of different vaccination choices justifies a cost evaluation for better investment which is worthy of healthcare systems and policymakers. Furthermore, though vaccination is inevitable to creating herd immunity against coronavirus and its variants of concerns, the providing and deciding about financial aspects of vaccination programs (the type of vaccine and the priorities) depending on each country are also critical.^25^

Hence, a cost‐effectiveness analysis (CEA) is needed to respond to related questions, including; is vaccination cost‐effective in coping with the pandemic? What are their economic benefits? Moreover, it should determine the cost of ending the pandemic through vaccine investments. Therefore, this study systematically reviews the economic evaluation studies regarding the economic evaluation of the Covid‐19 Vaccine. It hopes to provide useful economic information for health systems and help categorize their priorities regarding the type of vaccine for their country.

## 2 METHODS

### 2.1 Search strategy

In this study, required data was determined using Preferred Reporting Items for Systematic Reviews and Meta‐analyses (PRISMA) criteria.^26^ In this systematic review, Scopus, Medline/PubMed, Web of Science Core Collection, Cochrane Library, Google Scholar, Embase (Excerpta Medica Database) is a biomedical and pharmacological database produced by Elsevier databases were searched to identify articles related to the economic evaluation of Covid‐19 vaccine. All published studies up to March 2022 were included. To identify additional articles, the references mentioned in the main published articles were also searched.

The main keywords were included “SARS‐COV‐2,” “Coronavirus,” “Covid‐19,” “immunization,” “Vaccine,” “Economic evaluation,” “Cost utility,” “Cost‐effectiveness,” and “Cost benefit.”

### 2.2 Study selection

Independently, titles and abstracts identified in the articles were screened by two authors for obtaining the cost‐effectiveness reports of the Covid‐19 vaccine. Then, the full text of the selected studies was reviewed according to the inclusion and exclusion standards. Articles were screened by two authors and the results of each were compared. The unresolved discrepancies among reviewers were discussed and finalized by the third one.

### 2.3 Inclusion and exclusion criteria

The inclusion criteria based on the PICO protocol were consisted of:

1. Study design: economic assessment reports such as cost analysis, CEA, cost‐utility analysis (CUA), and cost‐benefit analysis (CBA)
2. Studied cases: vaccinated individuals against Covid‐19
3. Intervention: Types of Covid‐19 vaccines
4. Comparators: no vaccination
5. Outcome: any outcomes for economic evaluations ICER (incremental cost‐effectiveness ratio) as cost per life‐year gained (LYG), cost per case averted, cost per quality of adjusted‐life years (QALY), cost per Disability‐adjusted life years (DALYs), and net marginal benefit of interventions

Exclusion criteria

1. The study language other than English
2. The other types of studies such as the short reports, summaries, commentaries, conference abstracts, protocols, cost saving analysis, case reports or case series, editorials, letters and review articles
3. The literature was not available in full text

### 2.4 Quality assessment

In terms of methodology, the quality assessment of final articles was performed latest version of the Consolidated Health Economic Evaluation Reporting Standards CHEERS checklist (2022). This checklist consists of 28 questions to evaluate economic evaluation studies. Two independent reviewers sorted and graded one by one the selected articles in this review.^27^, ^28^ A decision on any disagreement was assigned and affirmed by a third arbitrator. Items that fully met the checklist in the selected studies were scored “1” and addressed as “Y.” Partially concordant cases received a score of “0.5” and were designated as “P,” and no concordant cases received a “zero” score and were designated as “N.” The high quality studies constituted >85% followed by very good quality <70%–85%, good <55%–70%, and low quality studies <55%.^29^, ^30^

## 3 RESULTS

### 3.1 Included studies

**Figure 1:**
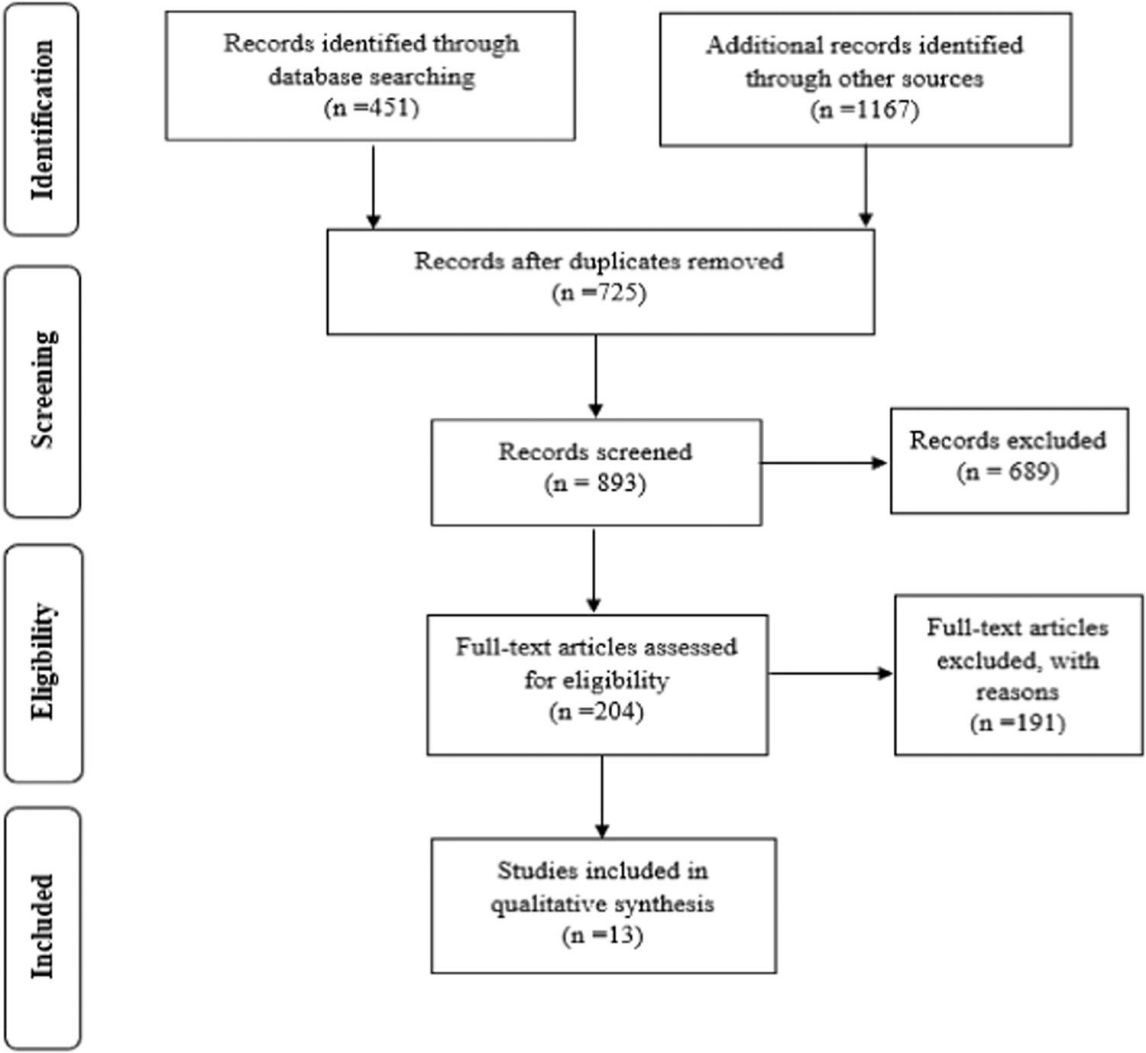
The procedure of systematic literature search strategy, it was in accordance with the preferred reporting items for systematic review.

In the initial assessment of this study 1618 associated citations were retrieved that 451 out of all were obtained from PubMed. Afterward, 725 duplicate citations were removed, and 893 studies based on their abstracts and titles remained. Next, 689 studies were excluded according to the inclusion standards and finally 204 abstracts were fixed for the full‐text screen. From all, 191publications were ignored considering the inclusion criteria and the CHEERS checklist was used to evaluate studies with inadequate results or methodology. Eventually, the presented systematic review was carried out on 13 selected studies. The review selection strategy based on the PRISMA guidelines was presented in Figure 1.

### 3.2 General characteristics of the studies

A total of 13 studies were reviewed according to included and excluded criteria. The studies were published between 2020 and the end of 2021. Studies have used various economic analyses to evaluate the Covid‐19 vaccine. One study used both cost‐utility and CBA,^31^ one CBA study,^32^ and one CUA study.^33^ One study also used both cost‐effectiveness and CBA.^34^ The other rest studies were of the CEA type. The studies have been performed in different countries. One study in Taiwan,^31^ one study from Brazil,^33^ one from Poland,^35^ one from Hong Kong,^36^ one from Turkey,^37^ and one study in six Western Pacific and southeast Asian countries,^38^ one study in Catalonia,^32^ three studies in the United States,^34^, ^39^, ^40^ one from Pakistan,^41^ one from Denmark^42^ and One study in Iran.^43^ Table 1 exhibits the characteristics of the studied articles.

**Table 1:** Characteristics of evaluated studies.

Study, year	Country	Type of study	Type of vaccine	Perspective	Time horizon	Outcomes	Intervention/comparator	Study model	Discount rate	Type of cost
Wang, 2020^31^	Taiwan	CBA, CUA	Pfizer, Moderna, AstraZeneca	Healthcare payer, societal	180 days	QALYs	Pfizer, Moderna, Oxford/No vaccination	Markov decision model	–	The direct cost of vaccine and COVID related medical cost, the indirect cost of productivity loss due to vaccine jabs and hospitalization
Fernandes, 2022^33^	Brazil	CUA	Oxford, CoronaVac, and Janssen	Public health system	1 year	QALYs	Oxford, CoronaVac, and Janssen/No vaccination	Markov models	–	Direct medical costs (medical visits, diagnostic tests, hospital stay (ward and ICU), hemodialysis, laboratory tests, imaging tests, and the unit cost of each vaccine dose)
Orlewska, 2021^35^	Poland	CEA	Comirnaty vaccine (BNT162b2)	Public healthcare payer	1 year	QALYs	Comirnaty vaccine (BNT162b2)/No vaccination	Markov models	EFF: 3.5%	Direct medical costs
Xiong, 2022^36^	Hong Kong	CEA	Sinovac, BioNTech	Healthcare sector	1 year	QALYs	Vaccination/No vaccination	Markov model	–	Direct medical costs (polymerase chain reaction tests, hospitalization care, and ICU care), cost of productivity loss
Hagens, 2021^37^	Turkey	CEA	–	Healthcare, societal perspective	1 year	DALY	Vaccination/No vaccination	Dynamic Transmission Model	Cost, EFF: 3%	Direct costs (care costs of hospitalization, the ICU stay, and pharmacotheraphy at home and vaccination) and indirect costs (production losses due to sickness leave and premature death)
Jiang, 2021^38^	Six Western Pacific and South East Asian countries	CEA	HB02, CoronaVac	Societal	1 year	QALYs	Vaccination/No vaccination	Decision tree models	Cost, EFF: 3%	Direct costs (vaccination program costs, medical treatment costs) and indirect costs (Productivity loss due to days spent in sickness and premature death before retirement)
López, 2021^32^	Catalonia	CBA	–	Social, health system	–	QALYs	Vaccination/No vaccination	Not mentioned	No discount rate	Direct costs and indirect costs
Padula, 2021^39^	United States	CEA	Sinovac, Biontech	Healthcare sector	1 year	QALYs	Vaccination/No vaccination	Markov mode	–	Direct costs, cost of productivity loss
Kohli, 2021^40^	United States	CEA	–	Healthcare system	–	QALYs	Vaccination/No vaccination	Markov mode	Cost: %3	Direct medical costs
Pearson, 2021^41^	Pakistan	CEA	–	Healthcare and partial societal perspectives	10‐year time horizon.	DALYs	Vaccination/No vaccination	Markov mode	Cost, EFF: 3%	Vaccination, testing, care and treatment costs, household costs incurred by COVID‐19 and patient management or costs of disease
Debrabant, 2021^42^	Danish	CEA	–	Healthcare sector	6 months	LY, QALY	Vaccination/No vaccination	Dynamic transition model	EFF: 2% or 4%	Direct costs, cost of productivity loss
Shaker, 2021^34^	United States	CEA, CBA	Universal vaccination versus risk‐stratified vaccination	Healthcare and partial societal perspectives	1 year	LY	COVID‐19 vaccination versus riskstratified vaccination approaches.	Decision tree	No discount rate	Direct costs and indirect costs
Vaezi, 2021^43^	Iran	CEA	CoronaVac, BBIBP‐CorV, mRNA‐1273, BNT162b2, rAd26‐S+ rAd5‐s, ChAdOx1nCoV‐19, Ad26.COV2.S	Healthcare and partial societal perspectives	Not mentioned	Averted death and DALY	Vaccination/No vaccination	Not mentioned	No discount rate	Direct costs and indirect costs

*Note*: QALY, quality‐adjusted life‐year; LY: life year; DALYs: disability‐adjusted life years; PSA: probability sensitivity analysis, CEA: cost‐effectivenenss analysis, CBA: cost‐benefit analysis; EFF: effectiveness; HB02: Sinopharm (HB02)‐associated vaccine name; BBIBP‐ CorV: The Sinopharm BIBP COVID‐19 vaccine name; BNT162b2: an mRNA‐based vaccine developed by Pfizer/BioNTech; rAd26‐S + rAd5‐s: The Janssen COVID‐19 vaccine; ChAdOx1: The ChAdOx1nCoV‐19 vaccine (AZD1222) was developed at Oxford University and consists of a replication‐deficient chimpanzee adenoviral vector ChAdOx1; Ad26.COV2.S: The Janssen COVID‐19 vaccine. ICER: incremental cost‐effectiveness ratio; NMB: net monetary benefit, Re: effective reproduction numbers, CN¥: Chinese yuan; NMB, net monetary benefit, BCR1: benefit—cost ratio payer perspective; BCR2: benefit—cost ratio societal perspective; ICUR: indicates incremental cost‐utility ratio.

Their perspectives has been defined as follows: two studies were from both healthcare and partial societal perspectives,^34^, ^41^ three studies from a healthcare sector perspective,^36^, ^39^, ^42^ one study from the healthcare system perspective,^40^ three studies from a healthcare payer perspective, and societal,^31^, ^32^, ^37^ a study from the perspective of the public health system,^33^ a study from the perspective of public healthcare payer,^35^ and a study from a societal perspective.^38^

In all studies except one study,^32^ the designed model of the studies was the Dynamic Transmission Model, Markov model, and Decision Tree. Two studies used a dynamic micro simulation model,^37^, ^42^ two studies used a Decision Tree^34^, ^38^ and seven studies used the Markov simulation model^31^, ^33^, ^35^, ^36^, ^39^, ^40^, ^41^ to extract long‐term data.

A time horizon is determined to review and follow up the interventions as well as their outcomes and costs in economic evaluation studies. In our selected studies, different time horizons are considered. There was a study with a time horizon of 180 days,^31^ another with 10 years,^41^ and also 6 months.^42^ Also, two studies did not mention the time horizon.^32^, ^40^ Other studies have had a 1‐year time horizon.

In different entered studies, discount rates have been dedicatedly assigned to alleviate costs and outcomes. For instance, 3.5% discount rate was applied in one study.^35^ In another study, a 3% discount rate was used to reduce costs and effectiveness.^37^, ^38^, ^41^ Two studies have also indicated that the discount rate has not been used due to the time horizon type.^32^, ^34^ In the study of Debrabant et al.,^42^ a discount rate of 2%–4% was used to discount the effectiveness.

Different health outcomes have been performed in the studies. In eight studies, only QALY^31^, ^32^, ^33^, ^35^, ^36^, ^38^, ^39^, ^40^ and in one study, only LY (life‐years) was used as a health outcome^34^; and one study used both QALY and LY,^42^ in two studies, DALYs was used to measure the health outcome.^37^, ^41^, ^43^

In some studies, the predominant intervention was vaccination versus non‐vaccination, but the type of vaccine was not mentioned.^32^, ^36^, ^37^, ^38^, ^39^, ^40^, ^41^, ^42^ In one of the studies, universal vaccination was compared with the Risk stratified vaccination approaches.^34^ In the study of Wang et al.,^31^ the intervention evaluated the vaccines of Pfizer, Moderna, and Oxford in comparison with non‐vaccination. In another study, the intervention evaluated Oxford, CoronaVac, and Janssen vaccines in comparison with non‐vaccination,^33^ and in another study, Comirnaty vaccine (BNT162b2) in comparison with non‐vaccination.^35^ In 5 of the 12 studies included, the type of vaccine was identified for analysis. One study looked at three vaccines, Pfizer, Moderna, and AstraZeneca,^31^ and another looked at three vaccines, Oxford, CoronaVac, and Janssen.^33^ Other studies have examined the Comirnaty vaccine (BNT162b2),^35^ Sinovac, BioNTech,^36^, ^39^ and HB02, CoronaVac,^38^ respectively.

In Table 2, the qualitative evaluation results of 13 studies are presented and it shows that all 13 studies passed the checklist and no study was excluded.

**Table 2:** CHEERS checklist.

Item	Item No	Wang	Fernandes	Orlewska	Xiong	Hagens	Jiang	López	*Padula*	*Kohli*	*Pearson*	Debrabant	Shaker	Vaezi
Title	1	Y	Y	Y	Y	Y	Y	Y	Y	Y	Y	Y	Y	Y
Abstract	2	Y	Y	Y	Y	Y	Y	Y	Y	Y	Y	Y	Y	Y
Background and objective	3	Y	Y	Y	Y	Y	Y	Y	Y	Y	Y	Y	Y	Y
Health economic analysis plan	4	Y	Y	Y	Y	Y	Y	Y	Y	Y	Y	Y	Y	Y
Study population	5	Y	Y	Y	Y	Y	Y	Y	Y	Y	Y	Y	Y	Y
Setting and location	6	Y	Y	Y	Y	Y	Y	Y	Y	Y	Y	Y	Y	Y
Comparators	7	Y	Y	Y	Y	Y	Y	Y	Y	Y	Y	Y	Y	Y
Perspective	8	Y	Y	Y	Y	Y	Y	Y	Y	Y	Y	Y	Y	Y
Time horizon	9	Y	Y	Y	Y	Y	Y	N	Y	Y	Y	Y	Y	N
Discount rate	10	Y	Y	Y	Y	Y	Y	Y	N	Y	Y	Y	Y	Y
Selection of outcomes	11	Y	Y	Y	Y	Y	Y	Y	Y	Y	Y	Y	P	Y
Measurement of outcomes	12	Y	Y	Y	Y	Y	Y	Y	Y	Y	Y	Y	P	Y
Valuation of outcomes	13	Y	Y	Y	Y	Y	Y	Y	Y	Y	Y	Y	Y	Y
Measurement and valuation of resources and costs	14	Y	Y	Y	Y	Y	Y	Y	Y	Y	Y	Y	Y	Y
Currency, price date, and conversion	15	Y	Y	Y	P	Y	Y	Y	Y	Y	Y	Y	Y	Y
Rationale and description of model	16	Y	Y	Y	Y	Y	Y	N	N	Y	Y	Y	Y	N
Analytics and Assumptions	17	Y	Y	Y	Y	Y	Y	Y	Y	Y	Y	Y	Y	Y
Characterizing heterogeneity	18	P	P	P	P	Y	P	P	P	P	Y	P	Y	P
Characterizing distributional effects	19	P	P	P	P	P	P	P	P	P	P	P	P	N
Characterizing uncertainty	20	Y	Y	Y	Y	Y	Y	N	Y	Y	Y	P	P	N
Approach to engagement with patients and others affected by the study	21	P	P	P	P	P	P	P	P	P	P	P	P	Y
Study parameters	22	Y	Y	Y	Y	Y	Y	Y	Y	Y	Y	Y	Y	Y
Summary of main results	23	Y	Y	Y	Y	Y	Y	Y	Y	Y	Y	Y	Y	Y
Effect of uncertainty	24	Y	Y	Y	Y	Y	Y	Y	Y	Y	Y	Y	Y	N
Effect of engagement with patients and others affected by the study	25	Y	Y	Y	Y	Y	Y	Y	Y	Y	Y	Y	Y	Y
Study findings, limitations, generalizability, and current knowledge	26	Y	Y	Y	Y	Y	Y	Y	Y	Y	Y	Y	Y	Y
Source funding	27	Y	Y	P	Y	Y	Y	Y	Y	Y	Y	Y	Y	Y
Conflict of interest	28	Y	Y	Y	Y	Y	Y	Y	Y	Y	Y	Y	Y	Y
Total percentage		26.5	26.5	26	26	27	26.5	23.5	24.5	26.5	27	26	25.5	22.5

*Note*: Y: completely presented (1 score), P: partially presented (0.5 score), N: no presented (0 score).

### 3.3 Cost outcomes

The included studies have reckoned different types of costs. All studies have calculated direct costs, including inpatient care, intensive care unit (ICU) care, diagnostic tests, other laboratory tests, imaging costs, treatment costs, home care services, the cost of each vaccine dose, and costs of the vaccination programs. Besides the direct costs, indirect costs, such as lost productivity costs have been calculated by eight studies.^31^, ^34^, ^36^, ^37^, ^38^, ^42^

Vaccination reduces the cost of treatment by reducing the number of infections detected. Reduced treatment costs following vaccination, in groups with a lower range of age, which have lower average treatment costs due to a lower risk of hospitalization (PLN 727 and PLN 872 in people 30–39 and 40–49 years, respectively) (PLN: average exchange rate in 2020: 1 EUR = 4.44 PLN), would be too negligible to reimbursed vaccine‐related activities.^35^

One of the studies estimated the cost of vaccination at €137 million, which is considerably negligible in comparison to the satisfactory results of a vaccination strategy at a calculated cost of €470 million. The largest share of the cost of the vaccination program was related to the reduction of ICU discharge cost (18%), and the share of the reduction in conventional hospital discharge (16%), reduction in (polymerase chain reaction test) PCR tests, and reduction of (rapid antigen test) RAT tests were 5% and 1%, respectively. Of the €137 million in vaccination costs, 13.5% (€37.26 million) was due to 2,854,806 doses of prescribed vaccine (average price €13.05) and 72% (€99.92 million) to total human resource costs, depreciation of infrastructures, and equipment.^32^

With 330 million population in the United States, without calculating the vaccination costs, the direct medical burden costs of the Covid‐19 epidemic on the healthcare sector have been estimated at about $34 billion in a year. The cost of the vaccination program has been estimated at $13,042 billion. The disease also caused 6.3 million days of hospitalization and more than 283,000 deaths, and the cost of reducing productivity lost due to the disease was estimated at $32 billion.^39^ In one study, the cost of healthcare was reduced from 2.4 million DKK (The Danish krone) to 1.5 million DKK when people were vaccinated. These reduction costs included hospital costs, testing costs, and follow‐up costs in Denmark (Table 3).^42^

**Table 3:** Summary results of included economic evaluation studies.

Study, year	Price(year)	Sensitivity analysis	Threshold	Health outcomes	Incremental cost	ICER	Is cost‐effectiveness?
Wang, 2021^31^	$2021	One‐way	Not mentioned	Moderna: 0.8284 Pfizer: 0.8119 AstraZeneca: 0.7456	ICUR (Moderna: −266.0500 Pfizer: −289.6303 AstraZeneca: −254.586) BCR (Moderna: 371.53 Pfizer: 351.33 AstraZeneca: 292.40)	ICUR (Moderna: −321.14 Pfizer: −356.75 AstraZeneca: −341.43) BCR (Moderna: 13.54 Pfizer: 23.32 AstraZeneca: 28.85)	Moderna and Pfizer vaccines won the greatest effectiveness among the three vaccines under consideration. all of the three vaccines dominated no vaccination strategy
Fernandes, 2022^33^	$2021	PSA	R$17586/QALY	Oxford: 0.002 CoronaVac: 0.0018 Janssen: 0.0008	Oxford: −47.46 CoronaVac: 32.41 Janssen: −17.77	Oxford: −23,161.3 CoronaVac: 17,757.85 Janssen: −1690.83	All the vaccines were cost‐effective
Orlewska, 2021^35^	PLN2020	One‐way	147,024	QALY lost/patient treated, death General: Population: 0.00763, 7.54 aged 30–39: 0.00741, 19.87 aged 40–49: 0.00746, 17.09 aged 60–69: 0.00838, 10.43 aged >80: 0.01157, 2.12	General Population: 1370.876 aged 30–39: 727.3 aged 40–49: 872.35 aged 60–69: 3536.628 aged >80: 13,632.02	General Population: 6249 aged 30–39: 67,823 aged 40–49: 28,135 aged 60–69: Cost saving aged >80: Cost saving	Vaccine was cost‐effective
Xiong, 2022^36^	HKD2021	one‐way & PSA	HKD 1,000,000/QALY	Before Omicron (no vaccine: 7,393,955 Vaccination program: 7,394,177) Omicron wave (no vaccine: 7,360,067 Vaccination program: 7,372,033)	Before Omicron (no vaccine: 0.84 (vaccination program: 5.80) Omicron wave (no vaccine: 5.1 Vaccination program: 8.8 HKD Billion)	Before Omicron: 22,339,700 Omicron wave: HKD 310,094	With increasing infection the vaccination program is cost effective and not cost‐effective until before the Omicron Prevalence
Hagens, 2021^37^	$2020	one‐way	Three‐times GDP per capita	No vaccination (QALYs lost:1,538,105) Vaccination (QALYs gained: 1,506,501)	No vaccination: 7,257,733,521 Vaccination (total incremental cost savings: 5,888,047,767)	Health Perspective: 511 Societal Perspective: Cost saving	Vaccination in Turkey is highly cost‐effective or even cost‐saving
Jiang, 2021^38^	2021 US$	One way and PSA	Once the GDP per capita	Hong Kong SAR, China: 105.18 Indonesia: 98.15 mainland China: 99.70 Philippines: 60.48 Singapore: 112.00 Thailand: 103.47	Hong Kong SAR, China: −40,255,438 Indonesia: −5,256,290 mainland China: −7,598,587 Philippines: −5,909,312 Singapore: −21,327,039 Thailand: −7,177,008	ICER (Hong Kong SAR, China, Indonesia, mainland China Philippines, Singapore, Thailand: dominant) NMB (Hong Kong SAR, China: 45,379,143 Indonesia: 5,662,212 mainland China: 8,645,824 Philippines: 10,196,996 Singapore: 28,632,981 Thailand: 7,984,741)	Inactivated COVID‐19 vaccines may be cost‐saving options
López, 2021^32^	€2021	–	Not mentioned	Social perspective: 116.67 System perspective: 19.93	137 million €	Social PERSPECTIVE (B/C ratio: 3.43) system perspective (B/C ratio health: 1.41	COVID‐19 vaccines is cost‐saving
Padula, 2021^39^	$2020	One way, PSA	$100,000/QALY	0.020	–16	Dominates	The vaccination program in Hong Kong was cost‐effective in the context of the Omicron
Kohli, 2021^40^	$2020	one‐way	$50,000 to $150,000	Not mentioned	No vaccine: $20,628 Vaccine: $27,718	50–64 years: $8200 18–49 years: $94,000	COVID‐19 vaccines is cost‐effective
Pearson, 2021^41^	$2020	one‐way	Pakistan does not have a fixed cost‐effectiveness threshold	70.1 DALYs	Health system perspective: $millions 2 societal perspectives: −20.2	Health system perspective: $27.9 per DALY societal perspectives: cost saving	COVID‐19 vaccines is cost‐effective
Debrabant, 2021^42^	2020 DKK	one‐way	Not mentioned	Discounted rate 2%: 1910 Discounted rate 4%: 1640	192,500	Vaccinated population 15%–25%: 128–796	Regarding the low vaccine prices, vaccinating a younger age groups may initially be cost‐effective
Shaker, 2021^34^	Not mentioned	One way, two‐way, PSA	$10,000,000	Universal vaccination: $17 Risk stratify: $19	Risk stratification: $116 Universal vs. risk stratified Per patient: −$57	Universal vaccination: $503,596,316 NMB (universal vaccination: $9,999,656 risk stratification: $9,999,395) ICER: ‐$65,518	Universal vaccination is cost‐effective
Vaezi, 2021^43^	$2021	No mentointed	Not mentioned	CoronaVac; 0.46, BBIBP‐CorV: 0.9, mRNA‐1273: 0.94, BNT162b2: 0.91, rAd26‐S + rAd5‐s: 0.82, ChAdOx1nCoV‐19: 0.74, Ad26.COV2S: 0.84	Not mentioned	CoronaVac; 121.2, BBIBP‐CorV: 39.3, mRNA‐1273: 37.0, BNT162b2: 20.8, rAd26‐S + rAd5‐s: 12.9, ChAdOx1nCoV‐19: 8.3, Ad26.COV2S: 6.2	Investing in COVID‐19 vaccine would be cost effective considering DALY

### 3.4 Uncertainty analysis

Except for one study,^32^ type of sensitivity analysis has been introduced in the other studies. A variety of one‐way, multiple, and probabilistic sensitivity analyzes have been performed to identify parameters that affect the stability of ICER results. Three studies of one‐way sensitivity analysis and probabilistic sensitivity analysis (PSA),^36^, ^38^, ^39^ one study of PSA,^33^ six studies of single‐way sensitivity analysis^16^, ^35^, ^37^, ^40^, ^41^, ^42^ and one study used all three types of sensitivity analysis one way, two‐way, PSA sensitivity analyzes.^34^

According to the results of the sensitivity analysis, some parameters are responsible for reducing the incremental cost‐utility ratio (ICUR), which include greater effectiveness of the vaccine, more daily vaccinations, more contagious of the disease, a lower proportion of the asymptomatic cases, a lower cost of the vaccine and prescribing, and higher medical costs. Regarding the common uncertainty of these parameters, large‐scale Covid‐19 vaccination by all three vaccine types resulted in nearly 100% cost savings (Pfizer, Moderna, and AstraZeneca) with a coverage rate of 70%.^31^ Based on the results of PSA in Brazil, ICUR decreases with age. And Oxford vaccine was dominant option compared to CoronaVac and Janssen for all groups of ages. Based on the payment threshold, all kinds of vaccines for patients over 60 years have been observed cost‐effectiveness; whereas CoronaVac and Janssen showed that it is not a true theory in subjects less than 59 years old.^33^ Moreover, the ICER was stricter to vaccine effectiveness, price, and SARS‐CoV‐2 infection rate in the general public and younger individuals.^35^ In another study in the Hong Kong, ICER decreased with increasing infection rate and gradually approached to the willingness‐to‐pay threshold of this country. Similar to the results of the Polish study,^35^ other authoritative variables on ICER rates, were vaccine price, vaccination rate, and hospitalization cost.^36^

Based on the results of sensitivity analysis of one study, one of the most influential parameters on the ICER rate was the number of susceptible people in the community. Also, a 2% increase or decrease in the cost of vaccination affected 10% on the ICER.^37^ In one study, the amount of net monetary benefit (NMB) also elevated along with the COVID‐19 incidence and vaccination implementation.^39^

In a study conducted in the United States, the results of a one‐way sensitivity analysis showed the strength of the cost‐effectiveness results by changing the parameters. Some parameters, such as vaccine cost, vaccination rate, and effectiveness of vaccine had the most significant influence on the results. The results of PSA also showed that at the threshold of willingness to pay $100,000/QALY, the vaccination option was cost‐effective.^40^

In another study in the United States, ICER results were highly sensitive to the parameter alterations in attack rate, vaccine price, and costs of hospitalizations; and the least effect on outcomes were related to the patient's disutility due to morbidity. According to the study, the target population‐specific vaccine should cost more than $150 per dose to exceed the incremental cost of more than $50,000 per QALY.^40^ As same as the other studies vaccine price, implementation costs, and plus the discount rate used to estimate LY & QALY gained affected the ICER results in the Danish study model.^42^ Global vaccination prevents 97,071 deaths and saves cost more than $17 billion. According to the results of the PSA, global vaccination with 99.58% would be the most cost‐effective strategy.^34^

### 3.5 Cost‐effectiveness outcomes

From a payer perspective, a dollar invested in the vaccine would result in a return of $2.79, $4.77, and $7.21 for Moderna, Pfizer, and AstraZeneca, respectively. Base on a community perspective, one dollar of investiture conducts to throwbacks of $6.05, $10.39, and $14.46 for Moderna, Pfizer, and AstraZeneca, respectively. In addition, the cost‐benefit ratio for all three vaccines increases when the value of statistical life and losses to the global economy and education are taken into account.^31^ All three vaccines were dominant against non‐vaccination with a coverage rate of 70%. The Moderna vaccine achieved an average of 0.8284 quality‐adjusted life days (QALDs) per person at a lower cost, indicating that the Moderna vaccine was a dominant strategy against vaccine absence (ICUR = 321.14). The Pfizer vaccine QALDs were similar to the Moderna vaccine ($31 per dose), but due to the cheaper price of the Pfizer ($14 per dose), more cost savings were observed compared to the Moderna (ICUR = −356.7512). For the AstraZeneca vaccine, the incremental QALDs were smaller than the other two vaccines (0.7456). Although it was cheaper than the other two vaccines (the US $5 per dose), it also had the lowest reduction in incremental costs (ICUR = 341.4381) due to higher demand for medical needs.^31^

The cost per QALY obtained related to the vaccination of the Polish general population was 6249 PLN. In age groups of 60–69 and over 80 years old, vaccination has been assigned as more effective and less expensive compared to no vaccination. And with vaccination in age groups of 40–49 and 30–39, the incremental cost per gained QALY was 28,135 and 67,823 PLN, respectively. Increased risk of hospitalization and hospitalization costs have a greater effect on ICER in the sub‐population of the 60–69 years compared to the youth age groups. Under the severe and acute conditions of the disease, except for those over 80, vaccination would not be known cost‐effective in all populations (Table 3).^35^

The vaccination program, which has reached about 72% of Hong Kong's population with two doses of the vaccine, cost the Hong Kong Dollar (HKD) 22,339,700 per QALY obtained before the Omicron wave and was not cost‐effective at the threshold of willingness to pay in this country. However, the cost‐effectiveness of the Covid‐19 vaccination program was highly sensitive to the infection rate. In the Omicron outbreak in this country, HKD 310,094 was determined for the vaccination program ICER, and the vaccination program in the Omicron outbreak has been cost‐effective.^36^ From a socially perspective Shaker study in Canada, despite some universal challenges to side effects of the vaccine like the anaphylaxis, global vaccination has been considered a cost‐effective approach unless the vaccine anaphylaxis risk was greater than 0.76%.^34^

## 4 DISCUSSION

Research has shown that primary mass vaccination of Covid‐19 is more prominent compared to doing nothing; as not only does it provide good financial value, it also saves more lives and QALY at a lower cost. The incremental cost per QALY gained by the adult population has been significant in different levels, compared with no vaccination worldwide.^39^, ^40^, ^42^ In probing on primary mass vaccination, researchers found that this strategy is effectiveness in reduction of hospitalization staying more than 50%, and in mortality rate by decreasing health costs to 90%. And at the threshold of the United State Dollar (USD) $50,000 as the wiliness‐to‐pay, primary mass vaccination probably was around 70% cost‐effective.^31^ It can be argued that different rates of cost‐effectiveness of vaccination programs between countries are because the background of the epidemic has varied between different regions, as well as how countries have dealt with the Covid‐19 outbreak and the extent of their control measures. The western countries, for example, generally had fewer strict control measures and the disease had been more prevalent. Therefore, as the epidemic intensifies and the rate of infection increases, the vaccine becomes more targeted and leads to fewer deaths, and less money lost.^38^

In recent years, due to the unexpected prevalence of Covid‐19 and the unpreparedness of human societies to deal with its adverse consequences such as death, tremendous expenditures have been spent on finding the most cost‐effective treatments, so most economic evaluations instead of preventable methods such as vaccinations have focused on the treatment of Covid‐19. And some treatments such as Remdesivir and Dexamethasone have shown cost savings and death prevented.^44^ As two studies have demonstrated that the administration of primary mass vaccines of Covid‐19 compared to its treatment methods in countries with different economic levels have remained a complementary cost‐saving and cost‐effective.^32^, ^39^ And assuming an epidemiological model with native data on demographics, social communication arrangements, costs of the healthcare system, gross domestic product (GDP), human productivity, and their salaries that has been mathematically modified into four sections including Susceptible, Infectious, Recovered, and Death; it could be applicable to conceptually representative for other countries as well.^32^ However to meet the global demand for vaccines, and conquer distributive challenges in the supply chain, the current vaccine production should be increased logistically; on another side vaccine prices are variable from country to country, and the beneficiary of vaccination may not be the same across the different populations. Therefore in this situation, the use of both vaccines and treatments is recommended by the researchers of the western countries.^41^

The results of some studies in the low‐middle‐, and high‐income countries in the western Pacific and Southeast Asia have shown that vaccination is not only cost‐effective but also has significant NMBs. This means that with the growth of prevalence of Covid‐19 and the population coverage of vaccination, the NMB will increase. And regarding the high population density and large population of these regions, protecting the population against COVID‐19 would be considered an important policy intervention. For example, the incremental cost‐effectiveness ratio (ICER) for a primary vaccination with Covid‐19 vaccines was estimated at 6.2–121.2 USD to avert one DALY and 566.8–10,957.7 USD per death in Iran.^40^, ^43^

According to one of the European studies, in terms of health policy, designating the vaccination movement has been known as a very effective strategy in restraining the disease; accounting, the whole of Catalonia and Spain, for savings of 227 and 1447 million euros from the health system perspective. By the direct effect of vaccination, the study estimates the avoided cases and recommends that a third dose of the vaccine not only preserves the cost‐benefit ratio but also would extend the duration of its positive effects; therefore, the results would indicate a lower threshold in the number of hospitalizations, ICU admissions, PCR, and RAT tests, and prevention mortality.^41^

To prospect the relative value of vaccines, and provide evidence‐based information to the American healthcare sector, policymakers, and generalize the findings to the other health systems around the world, some researchers based on the US economy using an economic model, despite a negative ICER at the threshold of $100,000/QALY, have predicted that existing vaccines can have a significant increase in effectiveness at a lower direct cost than doing nothing. And in their findings, the cost‐effectiveness acceptability curve showed cost‐effectiveness at threshold. However, the attack ratio for the year after vaccination is the disadvantage of using value. Prediction of mortality among common pattern is limited to a few months later, and changes in health policies or personal behavior may modify the long‐term certainty of the disease.^40^, ^41^

Estimates of some studies have shown that the value of vaccination in low‐risk groups (less than 50 years old) is significantly inferior to that value in older ages. Analyzing in this way makes some misunderstanding; for example, the group under 50, despite being at low risk of severe disease from Covid‐19, has shown the main role in spreading the disease in the communities, and their vaccination value has been underestimated as a preventable intervention for all the population; and in the other hand, many of the other societal costs such as their productivity associated with the pandemic has been refused to be noticed.^42^

On the other hand, there are different population stratifications in different studies estimating the vaccines effectiveness.^33^ Some of their evidence in Poland showed that in elderly, high‐risk, and vulnerable populations, vaccination was the first choice in controlling morbidity and mortality, especially in the early stages of vaccine administration.^35^ Although targeting older people in high‐income countries will initially be much more cost‐effective, some studies in low‐income countries have shown that these benefits are not particularly age‐dependent. In some low‐income countries such as Pakistan, individual vaccination would be cost‐effective if the vaccine production and delivery costs would be at $10 a dose or had an effectuality up to 30%; and despite the results of Taiwan's study,^31^ primary mass vaccination program in several years may evacuate insufficient resources from the other part of the Pakistan's health services, and could not be considered a cost‐effective intervention. Assuming a 1‐year vaccination in such countries, the cost would be a further $2 million in comparison with non‐vaccination, avoiding 70,000 DALYs, which would gain a $28 ICER per DALY. And assuming a 5–10‐year vaccination, it would have higher incremental costs ($228–454 per DALY, respectively) and more avoided DALY.^41^

As we mentioned above, vaccination in the age group under and above 50–60 had different ICERs in different countries, and when we included the productivity losses of the younger group and reduced the price of the vaccine, the efficiency and cost of the vaccine increased. In addition, the results showed that as the target group expanded, from the elderly to younger populations, an increase in cost per life year emerged. Based on the findings of Debrabant^42^ and their colleagues in Denmark, although we account for the ICER by the vaccine prices and hospitalization costs, this increasing cost may come from the marginal products of vaccination with its increasing costs; which pursues the low mortality rate of the Covid‐19 among those under 60 years free from hospitalization and vaccine costs.^42^ When comparing the risk stratification with global vaccine coverage, the researchers found that the risk of anaphylactic reaction was 2 in 500 people; and prompted healthcare officials to take extra precautions to vaccinate people with a history of all‐cause anaphylactic reactions in the United Kingdom, the United States, and Canada. A review of the Vaccine Adverse Events Registry System in 2019 showed that however, vaccine‐associated anaphylaxis is considerable, with a cumulative incidence rate of 0.0017%, it has been a treatable medical event for trained, experienced clinicians. Considering the factual ratio of 1.3 subjects suffered from anaphylaxis per million vaccine doses, the messenger RNA (mRNA) vaccines have been associated with an excess 0.1% anaphylaxis rate; that would be remarkably lower than that in comparison with the effective benefits and costs of receiving the Covid‐19 vaccine and its associated externalities for communities. A recently published recommendation from the American College of Allergy, Asthma, and Immunology Working Group on Covid‐19, instead of endorsing, does express a gradual grading vaccine method as a potential option for next dose vaccinations in individuals with a history of allergic response due to COVID‐19 vaccines or their constituents.^34^

To check the robustness of the cost‐utility or CEA of the Covid‐19 vaccine with variable values of parameters, including the reproduction number, and Monte Carlo simulations, the sensitivity analyses have been applied in some selected studies.^16^, ^37^, ^39^, ^41^, ^42^ And they showed that reproduction numbers ranged from 1.7 to 2.8 above one (lower bound to upper bound) in different rounds of the models in which the vaccination intervention was performed, and as a result, the healthcare costs were lower with the vaccination program than without vaccination. However, one of the studies^35^ showed that this parameter has affected only age group people (aged 20–49 years), and they have sustained the retransmission of SARS‐CoV‐2, with reproduction numbers much higher than one.

However, determining the method of vaccination against the Covid‐19 pandemic in any region or country is influenced by specific contextual factors of that place, including social conditions, economic level, the outlook of the healthcare system, and its share of GDP, and the transmission rate of the disease in that community. It requires agreement and cooperation between countries to acquire Covid‐19 vaccination and risk stratification determination and sharing. Although, implementation charges play a vital role in well‐timed access to significant promotions of the Covid‐19 vaccination and receiving its positive social externalities. Given the limitations of health budgets worldwide, evidence‐based decision‐making seems crucial; so, decision making based on the financial assessment of health interventions, especially cost‐effectiveness evaluation, is essential to create evidence‐based economic investigations to identify the most cost‐effective intervention against pandemics such as Covid‐19. The present study findings would have a significant contribution in notifying decision‐makers to refund health budgets, calculated the value of primary mass vaccination, preparing cost‐effective types of antiviral vaccination by using available products, and booster dose in new variants of the Covid‐19, appropriate allocating of expenditures, and distributing the vaccine, especially to low‐income nations strategically, and providing reliable information, and producing scientific evidence. Finally, the policy‐making choices regarding Covid‐19 vaccination results in to better economic management, efficiency improvements, and best allocation of health budget within the monetary limitations of the healthcare system.

### 4.1 Policy recommendations for future emerging infectious diseases

In this part, we attempt, through the findings of the selected studies, to suggest policy options to improve upstream motivations for policymakers as a platform for policy reformation that can decline future hazards of the emerging new infectious diseases. First of all, we need to consider that providing policy options is not a linear way, it requires a continuous process, which involves planning, reviewing, learning and improving. Arguably, we are trying to look at these global issues like the widespread infectious diseases from several dimensions, simultaneously at global, national, and subnational levels, including vaccine coverage, accessibility of vaccine, demands of vaccine doses, individual acceptance, and healthcare system requirements for vaccination. According to the reports received, by the end of the first month of 2022, of the 192 countries that used the Covid‐19 vaccine, among the five technology vaccine platforms with vaccines approved in one or more countries, as vaccine coverage, adenovirus vector vaccines are the most used (180 countries), followed by mRNA or messenger Ribo‐Nucleic Acid vaccines (159 countries), inactivated vaccines (116 countries), protein subunit vaccines (10 countries), and conjugate vaccines (3 countries).^45^ Based on the information of,^45^ from a regional perspective, both mRNA and adenovirus vector vaccines have been used in high‐income countries in Europe and America. While in most African countries, vaccination has mainly vectored with a combination of inactivated adenoviruses. In terms of access to vaccination,^46^ have shown that low‐income countries were less able to purchase the vaccine, despite global efforts such as the Global Access to Vaccine for Covid‐19 (COVAX). While most high‐income countries have purchased vaccines directly using advanced purchase agreements with vaccine developers and manufacturers. Regarding the demands for Covid‐19 vaccination, according to the reports of the WHO and the data population by the 2022, the highest demand for Covid‐19 vaccination was in Southeast Asia with 1.37 billion doses (population: 1,970,000,000), followed by the Western Pacific with 1.35 billion doses (population: 1,900,000,000), Africa with 1.16 billion doses (population: 1,120,161,000), the Americas with 0.88 billion doses (population: 1,010,000,000), Europe with 0.86 billion doses, and the Eastern Mediterranean with 0.78 billion doses. Although, vaccine‐induced immune responses in individuals with mild previous infection are generally higher,^47^ as previously infected individuals who were given one dose of a COVID‐19 vaccine have higher responses than full schedule vaccination of people who had not been previously infected.^48^, ^49^ Accordingly, it seems that for previously infected individuals a one‐dose plan might be adequate to reach the dual pursuit of covering populations and preserving stockpiles. Moreover, this statistic could be considered as the geographic distribution of the virus and also the primary measures that every region had taken place at the right time. On people acceptance, although we have seen varying degrees of vaccine hesitancy across countries, the impact appears to have been relatively small as acceptance of COVID‐19 vaccines is likely to change over time as stronger evidence and monetary incentives emerge. And, last but not least, healthcare system requirements for vaccination include executing adequate systems to document vaccine administration and activating effective reminder systems, providing immunization services without disparities by integrating healthcare services, logistics capacity, and workforce distribution.

Based on the retrograde picture of COVID‐19 vaccination policies, and conditional on the different countries' socio‐demographic index, healthcare access and quality Index, GDP per capita adjusted for purchasing power parity (PPP), and as well as PPP‐adjusted government health spending per capita, it is assessed that the disparity in vaccination coverage around the world implies that the susceptibility of unvaccinated populations in some countries may impede or boomerang pandemic management, especially in encountering new variants of COVID‐19 such as Omicron and other future emerging variants. Hence, more countries and organizations must be engaged in the global reaction to pandemics, take responsibility, enhance their national and regional accountability, and deliver leadership to overcome the complicated onrush challenges politically, technically, and financially.

### 4.2 Limitations

The present study limitations were as below:

1. There is a kind of uncertainty about the immunity time frame that has been produced by different types of vaccinations, and long‐time studies regarding the Covid‐19 vaccination efficacy are insufficient and demanded. It requires more research to support it.
2. Given the new virus variants emerging from the coronavirus such as the Omicron variant, only one of the included studies has been dealt with and its effectiveness has been proven by the authors of the article, but the effectiveness of other current vaccines can be doubted, so more research is needed to measure the cross‐protection from current vaccines on new variants of the virus.
3. Due to language limitations, our access is limited only to English studies and we could not use the studies conducted in other languages.

## 5 CONCLUSION

It would presumably be cost‐effective if the production of approved vaccines were further developed and applied worldwide. The prioritization of vulnerable target groups, such as high‐risk populations or the elderly, into productive populations, has generally posed challenges and critics in professional ethics. We suppose that if primary vaccination at a reasonable cost can reduce the transmission among the population with a higher chance and also prevent the transmission of the disease in the communities rather than just prevention of the disease, especially as a booster dose, its supply with the support of the world community in many low‐ and middle‐income countries will decline significantly the mortalities and morbidities' rate of Covid‐19 disease and hinder the boomerang effect for other countries with adequate measurements for the disease management. Although some studies have shown a significant economic correlation between the value of the vaccine and different age groups, especially in prioritized geriatrics groups, as well as booster vaccination in the context of increasing epidemics with new virus variants; it is suggested that due to the positive externalities of vaccination, primary mass vaccination, with the help of COVAX‐19TM, could be considered a reliable way to combat viral epidemics compared to the loss of individual lives and the economic and educational disturbances around the world.

## AUTHOR CONTRIBUTIONS

**Dolatshahi Zeinab**: Data curation; formal analysis. **Nargesi Shahin**: Resources; software; supervision; validation; visualization; writing—original draft; writing—review & editing. **Mezginejad Fateme**: Investigation; methodology; project administration. **Bagheri faradonbeh Saeed**: Conceptualization; data curation.

## CONFLICT OF INTEREST STATEMENT

The authors declare no conflicts of interest.

## ETHICS STATEMENT

Ethic approval has permitted by the ethical committee of Ilam University, Iran.

## TRANSPARENCY STATEMENT

The lead author Nargesi Shahin affirms that this manuscript is an honest, accurate, and transparent account of the study being reported; that no important aspects of the study have been omitted; and that any discrepancies from the study as planned (and, if relevant, registered) have been explained.

## Data Availability

As this is a systematic review, the data is public via databases to everyone. However, data is available from the corresponding author upon request.
